# Mitochondrial fission and fusion in *Dictyostelium discoideum:* a search for proteins involved in membrane dynamics

**DOI:** 10.1186/1756-0500-5-505

**Published:** 2012-09-14

**Authors:** Brixey G Schimmel, Gregory W Berbusse, Kari Naylor

**Affiliations:** 1 Biology Department, University of Central Arkansas, 180 Lewis Science Center, Conway, AR, 72035, USA

**Keywords:** Mitochondria, Fission, Fusion, Mitochondrial morphology, *Dictyostelium discoideum*, MidA, CluA, Dynamin

## Abstract

**Background:**

Mitochondrial morphology is maintained by two distinct membrane events -fission and fusion. Altering these conserved processes can disrupt mitochondrial morphology and distribution, thereby disrupting the organelle’s functionality and impeding cellular function. In higher eukaryotes, these processes are mediated by a family of dynamin-related proteins (DRP’s). In the lower eukaryotes, for instance *Dictyostelium discoideum,* mitochondrial fission and fusion have been implicated but not yet established. To understand the overall mechanism of these dynamics across organisms, we developed an assay to identify fission and fusion events in *Dictyostelium* and to assess the involvement of the mitochondrial proteins, MidA, CluA, and two DRP’s, DymA and DymB.

**Findings:**

Using laser scanning confocal microscopy we show, for the first time, that lower eukaryotes mediate mitochondrial fission and fusion. In *Dictyostelium,* these processes are balanced, occurring approximately 1 event/minute. Quantification of the rates in *midA*^-^, c*luA*^-^, *dymA*^*-*^, or *dymB*^*-*^ strains established that MidA appears to play an indirect role in the regulation of fission and fusion, while the DRP’s are not essential for these processes. Rates of fission and fusion were significantly reduced in c*luA*^-^cells, indicating that CluA is necessary for maintaining both fission and fusion.

**Conclusions:**

We have successfully demonstrated that *Dictyostelium* mitochondria undergo the dynamic processes of fission and fusion. The classical mediators of membrane dynamics - the DRP’s – are not necessary for these dynamics, whereas CluA is necessary for both processes. This work contributes to our overall understanding of mitochondrial dynamics and ultimately will provide additional insight into mitochondrial disease.

## Findings

### Background

Mitochondria are cellular organelles that produce energy in the form of ATP, play a central role in cellular metabolic pathways, and are essential for regulating apoptosis [1-3]. In most organisms, mitochondria are reticular, highly branched, complex structures [4]. The maintenance of this structure is vital for the success of these and other essential cellular functions. In both yeast and mammalian cells, this tubular structure is maintained by a balance of two dynamic and highly conserved processes: fission and fusion. Fission is the process by which mitochondria divide, while fusion occurs when neighbouring organelles join together to become one [1,4,5].

Disrupting these processes alters not only the mitochondrial morphology but also the distribution of mitochondria throughout the cell. Uneven distribution decreases the targeting efficiency of critical metabolites, such as ATP [6]. It has also been suggested that fission and fusion ensure a healthy population of mitochondria and protect against potential damage or loss of the mitochondrial DNA which contains the genes necessary to carry out cellular respiration [7]. These processes play a significant role in the regulation of apoptosis [2,8-10] and have been implicated in normal developmental pathways as well as in the progression of neurodegenerative diseases such as Charcot-Marie Tooth or dominant optic atrophy [2,11-13].

To understand the mechanism of these mitochondrial processes, studies have been undertaken in yeast and mammalian cells - the results of which indicate that mitochondrial fission and fusion are mediated by a family of dynamin-related proteins (DRP’s) [1,14-17]. DRP’s function in membrane remodelling events such as budding, organelle fission and fusion, endocytosis, and cytokinesis [18-21]. These proteins do not work alone; in mitochondria, multi-component machines—fission and fusion complexes—are responsible for remodelling the inner and outer mitochondrial membranes to allow a tubule to divide or fuse [5,17]. Although the processes themselves are highly conserved, variations in the protein composition of the complexes do occur. For example, mammalian cells lack the adaptors Mdv1 and Ugo1 that function in yeast cells [1,3,17,22].

In an effort to understand the overall mechanism of these processes and the differences between organisms, a study of the lower eukaryote, *Dictyostelium discoideum,* was undertaken. It has been suggested that *Dictyostelium* cells do carry out mitochondrial fission [23,24], but no actual fission or fusion events have been identified. Thus, we developed an *in vivo* assay to determine if *Dictyostelium* cells carry out the processes of mitochondrial fission and fusion and to identify potential molecular players in these pathways. *Dictyostelium* expresses a number of proteins that are associated (based on function, homology, or localization) with the mitochondria, including two DRP and three dynamin-like proteins [21]. For this study, we chose the two DRP’s, DymA and DymB, and two non-DRP’s, MidA and CluA. MidA was chosen because it does not induce a change in mitochondrial morphology yet plays a significant role in mitochondrial function [25]. CluA was chosen because deletion of this protein induces a clustered mitochondrial morphology comparable to a fission defect in yeast cells [26,27]. Here, we present the first time-lapse images showing actual fission and fusion events in *Dictyostelium* cells. We quantified these events to establish that mitochondrial fission and fusion are balanced processes in these cells. In addition, we have begun to determine which proteins are essential for these processes. Our results reveal that MidA and the DRP’s - DymA and DymB - are not required for mitochondrial fission or fusion. Interestingly, CluA appears to have some role in both processes as demonstrated by a decrease in both rates in the protein’s absence. The results from this work will further our knowledge of the mechanisms of fission and fusion, ultimately contributing to a better understanding of both membrane dynamics and, more importantly, mitochondrial disease.

## Methods

### Strain culture and growth conditions

All *Dictyostelium discoideum* strains described here were obtained from Dicty Stock Center [28], AX4 (wild-type) was deposited by Bill Loomis, *cluA*^*-*^ by Margaret Clarke, *dymA*^*-*^ and *dymB*^*-*^ by Dietmar Manstein, and *midA*^*-*^ by Ricardo Escalante. The strains were cultured axenically in liquid HL5 medium supplemented with streptomycin (Fisher) (final concentration 300 μg/ml) and ampicillin (Fisher) (final concentration 150 μg/ml) at 22 °C shaking at 125 rpm [29].

### Confocal microscopy of *Dictyostelium* mitochondria

AX4, *midA*^*-*^*,* and *cluA*^*-*^ cells were diluted to 3 X 10^4^ cells/ml in Lo-Flo liquid media (Formedium) until cells reached log phase (4–7 days). *dymA*^-^ and *dymB*^-^ cells were diluted into HL-5 instead of Lo-Flo. Log phase cells (5 ml) were washed by centrifuging at 500 x g for 4 minutes and resuspended in 5 ml of Lo-Flo (including *dymA*^-^ and *dymB*^*-*^*)* plus 1 μl of 1 mM MitoTracker Red CMXRos (Invitrogen) [30]. Cells were incubated at room temperature with shaking for 3–5 hours, washed twice with Lo-Flo, and resuspended in 5 ml Lo-Flo media. Washed and stained cells (0.5 ml) were placed in Lab TekII 4-well chambered coverglass (Nalge Nunc International) for imaging.

### Quantification of mitochondrial fission and fusion

Quantified cells were imaged on a Zeiss laser scanning LSM Pascal confocal microscope using a pinhole of 144 (1.36 airy units), resulting in an optical slice of 1.1 μm. A single plane was imaged every 677.38 milliseconds for 100 seconds or until bleaching occurred. Fission and fusion events were identified in a minimum of 35 cells for each strain. Fission was identified when one organelle became two if, prior to this, no out-of-focus mitochondria had been visible. Fusion was identified when two mitochondria rotated around one another for a couple of frames (They never resolved into one during this time.) then became one. If two organelles came together, or split apart and were in their original state by the next frame, this was classified as a “drive by” and not quantified. Rates were calculated by averaging the number of events/min/cell for each strain and are presented as mean ± standard error. Statistical analysis was performed using JMP 9.0.0 (SAS Institute, Inc) software. Wilcoxon rank-sum nonparametric t-tests were conducted for statistical comparisons of fission and fusion within and among strains; p-values less than 0.05 were considered statistically significant.

To be as stringent as possible with this assay, the data presented here has been repeated independently by a second individual who obtained statistically similar results (data not shown).

In an effort to confirm that the identified fission and fusion events are not from mitochondria entering and leaving the focal plane, small z-stacks were imaged. These included 3 planes of 1.1 μm depth, images were flattened and quantified as above.

## Results

### *Dictyostelium* mitochondria undergo fission and fusion

**Figure 1:**
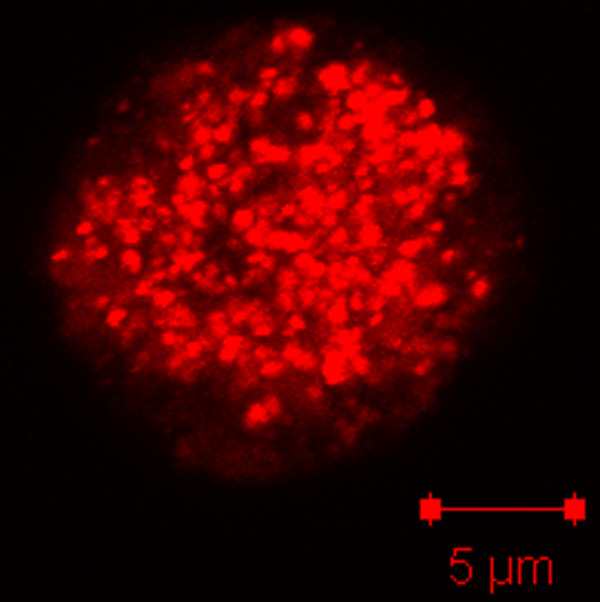
**Projection of a* Dictyostelium *cell with fluorescent mitochondria.*** Dictyostelium * mitochondria are numerous and spherical in structure. The image is a projection of 13 z-plane confocal images, using a 63X objective.

Recent work in the model organism, *Dictyostelium discoideum,* has suggested that the process of mitochondrial fission takes place; however, fixed-cell studies have conclusively demonstrated that *Dictyostelium* mitochondria are spherical organelles rather than the well studied tubular mitochondria found in most other organisms [20,21,23,24]. The simplicity of these spherical mitochondria suggests that the processes of fission and fusion are not necessary to maintain this organelle morphology. Thus, to determine if mitochondrial fission and fusion actually occurs in *Dictyostelium* cells, we developed a live cell fission and fusion assay. Upon first analysis of the mitochondria, it was apparent that there were too many organelles to clearly distinguish fission and fusion events in a whole cell (Figure 1). 

**Figure 2:**
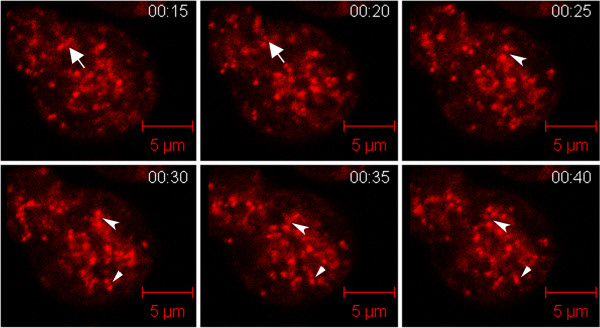
**Montage of* Dictyostelium * mitochondria over time. ***Dictyostelium* mitochondria undergo fission and fusion. The arrow points to a fission event, the arrowhead indicates a fusion event, and the concave arrow indicates a hotspot that undergoes first fusion and then fission. These single plane images were acquired every 5 seconds with a 0.8 μm optical depth by a 63X objective. Please note: this image was not used for quantification of fission and fusion event as the time points are too far apart.

**Figure 3:**
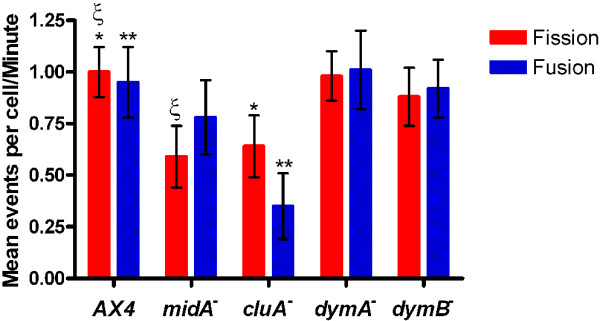
**Rates of fission and fusion in wild-type, *midA***^**-**^**, *cluA***^***-***^**, *dymA***^***-***^**, and *dymB***^***-***^** strains.** In all strains, the rates of fission and fusion were balanced (α = 0.05 for all comparisons). Symbols indicate significant differences between the rates of fission/fusion. Wild-type mitochondria undergo fission and fusion approximately one event/min. *midA*^*-*^ cells have a significantly decreased fission rate (p-value = 0.0056) while the fusion rate is statistically no different (p-value = 0.17) than wild-type. Fission and fusion rates in *cluA*^*-*^ cells are significantly decreased (p-value = 0.0218 and 0.0001 respectively) from wild-type, occurring approximately 1 event every 2 minutes. *dymA*^*-*^ and *dymB*^*-*^ cells do not significantly differ from wild-type cells in terms of mitochondrial fission and fusion (*dymA*^*-*^ vs. AX4 fission: p-value = 0.93; fusion: p-value = 0.934; *dymB*^*-*^ vs. AX4 fission: p-value = 0.38; fusion: p-value = 0.99).

Using laser scanning confocal microscopy with a pinhole of 1.35 airy units, which produces a 1.1 μm optical depth and Mitotracker Red (a well established mitochondrial marker) [30], a single plane of each cell was observed for fission or for fusion. The results demonstrated that *Dictyostelium* mitochondria do indeed undergo both of these dynamic processes (Figure 2, Additional file 1). Upon quantification of these events, it was apparent that, like yeast, the average rates of fission and fusion were balanced (p-value = 0.40), occurring at a rate of 1.00 ± 0.12 (mean ± standard error) fission events/min and 0.95 ± 0.17 fusion events/min (Figure 3). In order to ensure that identified fission and fusion events were not the result of organelles travelling into or out of the focal plane, small z-stacks (3 image planes of 1.1 μm each) were imaged. Quantification of these flattened z-stacks indicated that fission and fusion still took place and were balanced; thus, the identified events in the single plane imaging were genuine and not the result of mitochondria travelling through the focal plane. 

### MidA is not involved in mitochondrial fission or fusion

There are numerous proteins, based on function or homology, which may play a role in mitochondrial fission or fusion. We chose four of these proteins and used our assay to determine if they were involved in these processes. MidA is a putative methyltransferase that is targeted to the mitochondria and is required for mitochondrial complex I function [31]; it has been identified in *Dictyostelium* and humans [25] with a possible homolog in budding yeast [32]. Mitochondrial morphology of *midA*^-^ cells is not altered from wild-type [25]; thus, we did not expect MidA to be involved in fission or fusion. Interestingly, when the processes were quantified in *midA*^-^ cells, the fission rate (0.59 ± 0.15 events/min) was significantly decreased as compared to the wild-type (p-value = 0.0056). In contrast, the average rate of fusion (0.78 ± 0.18 events/min) was not statistically different from wild-type (p-value = 0.17). Despite the decrease of fission, the rates between the two processes are balanced (p-value = 0.527) and there is no resulting morphological change so, most likely, MidA is not directly involved in mitochondrial fission (Figure 3, Additional file 2).

### CluA is necessary for maintaining rates of fission and fusion

**Figure 4:**
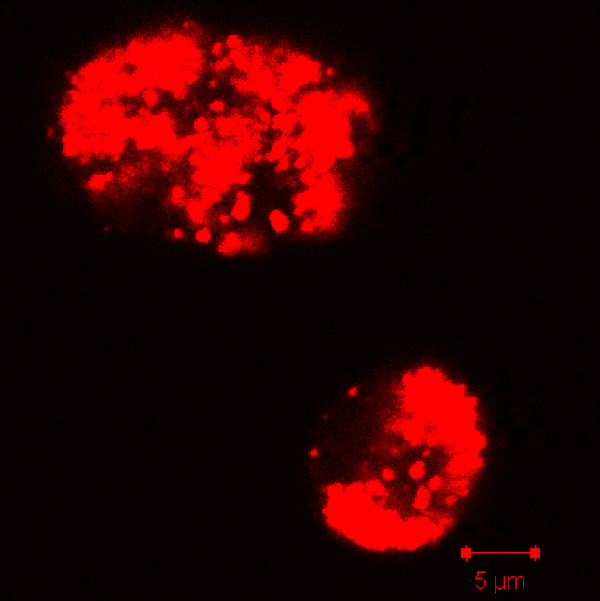
***cluA***^***-***^** cells have clustered mitochondria.** Live single plane image of *cluA*^*-*^ cells demonstrates a clustered morphology. Image was acquired with a 1.1 μm optical depth with no averaging by a 63X objective.

Homologs of the *Dictyostelium* protein, CluA, have been identified in *Arabidopsis thaliana, Caenorhabditis elegans, Drosophila melanogaster,, Homo sapiens, and Saccharomyces cerevisiae*. Mutations of this protein result in clustered mitochondria in all organisms with the exception of *C. elegans* (Figure 4) [27,33,34]. In *cluA*^*-*^cells, fission and fusion rates remained balanced, with 0.64 ± 0.15 fission events/min and 0.35 ± 0.16 fusion events/min (p-value = 0.079), but were significantly decreased as compared to wild-type cells (fission: p-value = 0.0218; fusion: p-value = 0.0001) (Figure 3, Additional file 3). Interestingly, the fission and fusion events were only found among mitochondria not a part of a cluster. For example in Figure 4 and Additional file 3, fission and fusion can be seen in the top, less clustered cell but none were found in the bottom cell (Additional file 3). These results suggested that CluA is necessary to maintain the rates of mitochondrial dynamics at the level found in wild-type cells. 

### Classical DRP’s are not essential for mitochondrial dynamics in *Dictyostelium*

**Figure 5:**
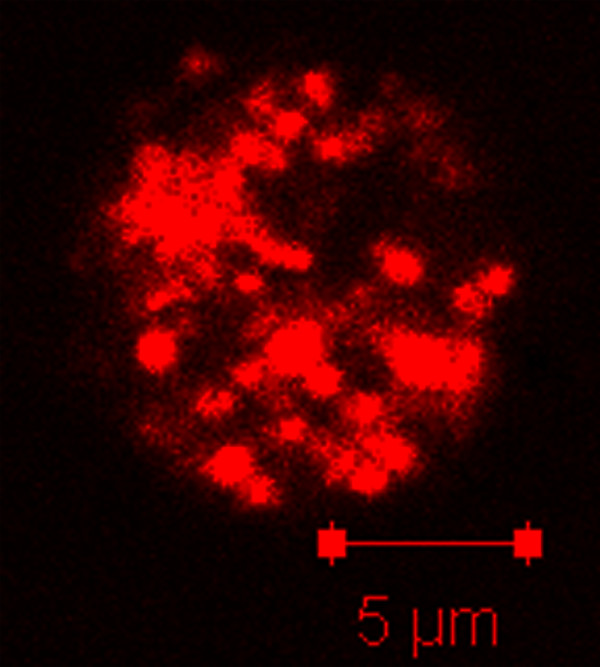
*** dymA***^***-***^** cells have aggregated mitochondria.** Live single plane image of *dymA*^*-*^ cells demonstrates a slightly aggregated morphology, along with some tubules. Image was acquired with a 1.1 μm optical depth with no averaging by a 63X objective.

Here we analyzed the two DRP’s, dynamin A (DymA) and dynamin B (DymB), for function in mitochondrial dynamics. Cells lacking DymA exhibit loose aggregates of both spherical and tubular mitochondria (Figure 5) [20]. Analysis of *dymA*^*-*^ cells showed that the mitochondria have an average fission rate of 0.98 ± 0.12 events/min while fusion occurs at an average rate of 1.01 ± 0.19 events/min (Figure 3, Additional file 4). These rates were balanced (p-value = 0.82) and were not significantly different from that observed in wild-type cells (fission: p-value = 0.93; fusion: p-value = 0.934). Thus, these results suggest that DymA is not essential for fission or fusion of the mitochondria in *Dictyostelium* cells. 

DymB transiently localizes to the mitochondria - perhaps to complete post-translational processing- although deletions of DymB do not alter mitochondrial morphology [21]. In an effort to determine if DymB functions in mitochondrial dynamics, *dymB*^*-*^ mitochondria were examined and the rates of fission and fusion were found to be balanced (p-value = 0.83) and occur at a rate of 0.88 ± 0.14 fission events/min and 0.92 ± 0.14 fusion events/min (Additional file 5). These rates are not significantly different from AX4 (fission: p-value = 0.38; fusion: p-value = 0.99), therefore DymB is also not required for mitochondrial fission or fusion.

## Discussion

Here, we presented our findings on the processes of fission and fusion in *Dictyostelium* cells. Using our *in vivo* quantification assay, we demonstrated for the first time that, although *Dictyostelium* mitochondria are spherical in structure, their mitochondria do undergo fission and fusion. In further support of this result, *Dictyostelium* mitochondria morphology is very similar in appearance to peroxisomes. Peroxisomes have been shown to undergo the dynamic process of fission (though not fusion) [35]; thus, even spherical organelles require some dynamics. Based on the spherical structure, one might expect fission to occur more often than fusion to create the spheres rather than tubules that are found in other organisms. In *Dictyostelium*, the events are balanced, occurring approximately 1 event/min; interestingly, this is about twice as often as the rates published for yeast [5].

In an effort to begin identifying potential players in the fission and fusion processes, we examined four proteins that have some known association with the mitochondria. MidA appears to significantly alter mitochondrial function by disrupting the assembly of complex I in the electron transport chain [31]. Mutations in this protein result in numerous deleterious phenotypes ranging from small cell size and slow growth rate to a decrease in phagocytosis and macropinocytosis as well as a 70% reduction in ATP production [25].

Although fission and fusion are slightly decreased (fission more so than fusion), the rates are still balanced and mitochondrial morphology is not altered. This decrease in mitochondrial dynamics may be an indirect result of the inadequately functioning mitochondria. The work presented here suggests that MidA does not play a direct role in the processes of fission and fusion.

Transmission electron microscope images of *cluA*^*-*^ cells indicate that the mitochondria are connected to one another, forming the characteristic cluster [24]. Our results demonstrate that there is a significant decrease in both fission and fusion processes of these mitochondria, resulting in a fission/fusion event approximately every two minutes. These collective results suggest that perhaps in the absence of CluA fission and/or fusion may be initiated but not completed, resulting in a connected cluster of organelles. The inability to complete the initiated events may be due to a loss of interaction with the cytoskeleton. CluA has homology to the protein-protein interaction region of the kinesin light chain - the tetratricopeptide repeats (TPR) domain [24] - suggesting that CluA could interact with kinesin and thus, the cytoskeleton. Studies performed in flies and plants support this conclusion [33,34]. However, there are many TPR proteins that do not interact with kinesin; an example is Fis1, a TPR protein which is required for mitochondrial fission in yeast and mammalian cells [36]. Ultimately, to tease out the specific role CluA plays in these processes, further studies will need to be pursued; for example, identifying interacting partners of CluA will help to determine if it connects to the cytoskeleton and/or to other potential fission and fusion proteins.

Finally, we looked at the classical proteins that mediate membrane remodelling events, the DRP’s. Previous work suggests that both DymA and DymB have primary functions elsewhere in the *Dictyostelium* cell - endosomal pathway and vacuole/peroxisome dynamics respectively [20,21]. However, several proteins have been co-opted to work in multiple organelle remodeling events. In yeast for instance, both the peroxisome and the mitochondria use the DRP, Dnm1, to carry out fission [37]. Thus, we analyzed both DymA and DymB to see if one of these proteins may have been co-opted to work in multiple remodelling events. *dymA*^*-*^ and *dymB*^*-*^ mitochondria undergo fission and fusion about once every minute (equivalent to the rate found in wild-type cells) thus, DymA and DymB are not essential for these processes and have not been co-opted to mediate multiple organelle remodelling events. Although DRP’s have been found to be responsible for mediating fission and fusion in all organisms studied to date, this is the first time it has been demonstrated that the classical DRP’s are not required for mitochondrial dynamics. It is possible that the DRP-like proteins play this role in *Dictyostelium* or more likely the FtsZ homologs, FszA and FszB, as suggested by Gilson et al., 2003 [23].

## Conclusions

In summary, we have shown for the first time that *Dictyostelium* mitochondria undergo fission and fusion like all organisms studied to date. The rates of these events are balanced, occurring at a rate of approximately 1 event/min. MidA is not essential for these processes nor are the classical DRP’s, DymA and DymB, while CluA is necessary for both fission and fusion. This work contributes to our understanding of the overall mechanism of mitochondrial fission and fusion, but it is lacking the identification of the proteins specifically involved in these processes. Our goal is to use this assay to continue the search for these proteins, specifically asking if FszA or FszB might be responsible for mediating mitochondrial fission or fusion in place of the DRP’s.

### Availability of supporting data

The data sets supporting the results of this article are included within the article and its additional files.

## Abbreviations

DRP, dynamin-related protein; DymA, dynamin A; DymB, dynamin B; TPR, tetratricopeptide repeats.

## Competing interests

The authors declare that they have no competing interests.

## Authors’ contributions

BGS participated in image acquisition, quantification of mitochondrial dynamics and statistical analysis. GWB obtained and established all strains used in this study and participated in quantification of mitochondrial dynamics. KN conceived and designed the study and participated in image acquisition. All authors took part in drafting the manuscript and have read and approved the final manuscript.

## Supplementary Material

Additional file 1 **MPEG (.mp4) Live cell movie of* Dictyostelium *mitochondria.** Mitochondria in *Dictyostelium* cells are very dynamic, rapidly moving and undergoing fission and fusion. The circle indicates a fusion event occurring at 15–16 seconds, the box indicates a fission event occurring at 8–10 seconds. Single plane images were acquired every 677.38 milli-seconds with a 1.1 μm optical depth by a 63X objective. The movie is presented at 2 frames per second. Click here for file

Additional file 2 **MPEG (.mp4) Live cell movie of* midA***^***-***^** mitochondria.** Mitochondria in *midA*^*-*^ cells are dynamic, undergoing fission and fusion, though slightly slower than wild-type cells. The circle indicates a fusion event occurring at 15–16 seconds, the box indicates a fission event occurring at 14–18 seconds. Single plane images were acquired every 677.38 milli-seconds with a 1.1 μm optical depth, no averaging and a 63X objective. The movie is presented at 5 frames per second. Click here for file

Additional file 3 **MPEG (.mp4) Live cell movie of* cluA***^***-***^** mitochondria.** Mitochondria in *cluA*^*-*^ cells are clustered and have significantly decreased dynamics. The circle indicates a fusion event occurring at 40–42 seconds, the box indicates a fission event occurring at 48–52 seconds. Note: within the circle there are a series of fission and fusion events, but they are several frames apart. Single plane images were acquired every 677.38 milli-seconds with a 1.1 μm optical depth, no averaging and a 63X objective. The movie is presented at 3 frames per second. Click here for file

Additional file 4 **MPEG (.mp4) Live cell movie of* dymA***^***-***^** mitochondria.** Mitochondria in *dymA*^*-*^ cells are slightly aggregated, may form tubules, or align into tubule like structures. Dynamics are similar to wild-type cells. The circle indicates a fusion event occurring at 22–24 seconds; the box indicates a fission event occurring at 1–5 seconds. Note: within the square, the movie begins a with fusion event distinctly separated by several frames before the fission event. Single plane images were acquired every 677.38 milli-seconds with a 1.1 μm optical depth, no averaging and a 63X objective. The movie is presented at 3 frames per second. Click here for file

Additional file 5 **MPEG (.mp4) Live cell movie of * dymB***^***-***^** mitochondria.** Mitochondria in *dymB*^*-*^ cells are morphologically the same as wild-type cells with similar dynamics. The circle indicates a fusion event occurring at 1:09–1:12 minutes; the box indicates a fission event occurring at 12–14 seconds. Single plane images were acquired every 677.38 milli-seconds with a 1.1 μm optical depth, no averaging and a 63X objective. The movie is presented at 5 frames per second. Click here for file

## References

[B1] Osteryoung KW Nunnari J The division of endosymbiotic organelles Science 2003 302 5651 1698 1704 10.1126/science.1082192 14657485

[B2] Karbowski M Youle RJ Dynamics of mitochondrial morphology in healthy cells and during apoptosis Cell Death Differ 2003 10 8 870 880 10.1038/sj.cdd.4401260 12867994

[B3] Scott I Youle RJ Mitochondrial fission and fusion Essays Biochem 2010 47 85 98 10.1042/bse0470085 20533902 PMC4762097

[B4] Johnson LV Walsh ML Chen LB Localization of mitochondria in living cells with rhodamine 123 Proceedings of the National Academy of Science, USA 1980 77 990 994 10.1073/pnas.77.2.990 PMC348409 6965798

[B5] Nunnari J Marshall W Straight A Murray A Sedat JW Walter P Mitochondrial transmission during mating in S. cerevisiae is determined by mitochondrial fusion and fission and the intramitochondrial segregation of mtDNA Molecular Biology of the Cell 1997 8 7 1233 1242 9243504 10.1091/mbc.8.7.1233 PMC276149

[B6] Chen H Chan DC Mitochondrial dynamics–fusion, fission, movement, and mitophagy–in neurodegenerative diseases Human Molecular Genetics 2009 18 R2 R169 R176 10.1093/hmg/ddp326 19808793 PMC2758711

[B7] Detmer SA Chan DC Functions and dysfunctions of mitochondrial dynamics Nat Rev Mol Cell Biol 2007 8 11 870 879 10.1038/nrm2275 17928812

[B8] Karbowski M Lee YJ Gaume B Jeong SY Frank S Nechushtan A Santel A Fuller M Smith CL Youle RJ Spatial and temporal association of Bax with mitochondrial fission sites, Drp1, and Mfn2 during apoptosis J Cell Biol 2002 159 6 931 938 10.1083/jcb.200209124 12499352 PMC2173996

[B9] Frank S Gaume B Bergmann-Leitner ES Leitner W Robert EG Catez F Smith CL Youle RJ The role of dynamin-related protein 1, a mediator of mitochondrial fission, in apoptosis Developmental Cell 2001 1 515 525 10.1016/S1534-5807(01)00055-7 11703942

[B10] Jin C Reed JC Yeast and apoptosis Nat Rev Mol Cell Biol 2002 3 6 453 459 10.1038/nrm832 12042767

[B11] Zuchner S Mersiyanova IV Muglia M Bissar-Tadmouri N Rochelle J Dadali EL Zappia M Nelis E Patitucci A Senderek J Parman Y Evgrafov O Jonghe PD Takahashi Y Tsuji S Pericak-Vance MA Quattrone A Battologlu E Polyakov AV Timmerman V Schroder JM Vance JM Mutations in the mitochondrial GTPase mitofusin 2 cause Charcot-Marie-Tooth neuropathy type 2A Nat Genet 2004 36 5 449 451 Epub 2004 Apr 2004 10.1038/ng1341 15064763

[B12] Delettre C Lenaers G Griffoin J-M Gigarel N Lorenzo C Belenguer P Pelloquin L Grosgeorge J Turc-Carel C Perret E Astarie-Dequeker C Lasquellec L Arnaud B Ducommun B Kaplan J Hamel CP Nuclear gene OPA1, encoding a mitochondrial dynamin-related protein, is mutated in dominant optic atrophy Nature Genetics 2000 26 2 207 210 10.1038/79936 11017079

[B13] Alexander C Votruba M Pesch UEA Thiselton DL Mayer S Moore A Rodriguez M Kellner U Leo-Kottler B Auburger G Bhattacharya SS Wissinger B OPA1, encoding a dynamin-related GTPase, is mutated in autosomal dominant optic atrophy linked to chromosome 3q28 Nature Genetics 2000 26 2 211 215 10.1038/79944 11017080

[B14] Otsuga D Keegan BR Brisch E Thantcher JW Hermann GJ Bleazard W Shaw J The dynamin GTPase, Dnm1p, controls mitochondrial morphology in yeast Journal of Cell Biology 1998 143 333 349 10.1083/jcb.143.2.333 9786946 PMC2132834

[B15] Bleazard W McCaffery JM King EJ Bale S Mozdy A Tieu Q Nunnari J Shaw JM The dynamin-related GTPases, Dnm1, regulates mitochondrial fission in yeast Nature Cell Biology 1999 1 298 304 10.1038/13014 PMC3739991 10559943

[B16] Sesaki H Jensen RE Division versus fusion: Dnm1p and Fzo1p antagonistically regulate mitochondrial shape Journal of Cell Biology 1999 147 4 699 706 10.1083/jcb.147.4.699 10562274 PMC2156171

[B17] Shaw JM Nunnari J Mitochondrial dynamics and division in budding yeast Trends in Cell Biology 2002 12 4 178 184 10.1016/S0962-8924(01)02246-2 11978537 PMC3785940

[B18] Osteryoung KW Organelle fission in eukaryotes Curr Opin Microbiol 2001 4 6 639 646 10.1016/S1369-5274(01)00263-6 11731314

[B19] Sever S Dynamin and endocytosis Curr Opin Cell Biol 2002 14 4 463 467 10.1016/S0955-0674(02)00347-2 12383797

[B20] Wienke DC Knetsch MLW Neuhaus EM Reedy MC Manstein DJ Disruption of a Dynamin Homologue Affects Endocytosis, Organelle Morphology, and Cytokinesis in Dictyostelium discoideum Mol Biol Cell 1999 10 1 225 243 9880338 10.1091/mbc.10.1.225 PMC25165

[B21] Rai A Nothe H Tzvetkov N Korenbaum E Manstein D Dictyostelium dynamin B modulates cytoskeletal structures and membranous organelles Cellular and Molecular Life Sciences 2011 68 2751 2767 10.1007/s00018-010-0590-5 21086149 PMC3142549

[B22] Scott SV Cassidy-Stone A Meeusen SL Nunnari J Staying in aerobic shape: how the structural integrity of mitochondria and mitochondrial DNA is maintained Curr Opin Cell Biol 2003 15 4 482 488 10.1016/S0955-0674(03)00070-X 12892790

[B23] Gilson PR Yu X-C Hereld D Barth C Savage A Kiefel BR Lay S Fisher PR Margolin W Beech PL Two Dictyostelium Orthologs of the Prokaryotic Cell Division Protein FtsZ Localize to Mitochondria and Are Required for the Maintenance of Normal Mitochondrial Morphology Eukaryotic Cell 2003 2 6 1315 1326 10.1128/EC.2.6.1315-1326.2003 14665465 PMC326642

[B24] Fields SD AQ Heuser J Clarke M Mitochondrial membrane dynamics are altered in cluA- mutants of Dictyostelium Journal of Muscle Research and Cell Motility 2002 23 829 838 10.1023/A:1024492031696 12952081

[B25] Torija P Vicente JJ Rodrigues TB Robles A Cerdan S Sastre L Calvo RM Escalante R Functional genomics in Dictyostelium: MidA, a new conserved protein, is required for mitochondrial function and development Journal of Cell Science 2006 119 6 1154 1164 10.1242/jcs.02819 16507593

[B26] Fields SD Conrad MN Clarke M The S. cerevisiae CLU1 and D. discoideum cluA genes are functional homologues that influence mitochondrial morphology and distribution Journal of Cell Science 1998 111 12 1717 1727 9601101 10.1242/jcs.111.12.1717

[B27] Zhu Q Hulen D Liu T Clarke M The cluA- mutant of Dictyostelium identifies a novel class of proteins required for dispersion of mitochondria Proceedings of the National Academy of Sciences 1997 94 14 7308 7313 10.1073/pnas.94.14.7308 PMC23817 9207087

[B28] Dictybase www.dictybase.org

[B29] Fey P Kowal AS Gaudet P Pilcher KE Chisholm RL Protocols for growth and development of Dictyostelium discoideum 2007 2 6 1307 1316 10.1038/nprot.2007.178 17545967

[B30] Poot M Zhang YZ Krämer JA Wells KS Jones LJ Hanzel DK Lugade AG Singer VL Haugland RP Analysis of mitochondrial morphology and function with novel fixable fluorescent stains Journal of Histochemistry & Cytochemistry 1996 44 12 1363 1372 10.1177/44.12.8985128 8985128

[B31] Carilla-Latorre S Gallardo ME Annesley SJ Calvo-Garrido J Grana O Accari SL Smith PK Valencia A Garesse R Fisher PR Escalante R MidA is a putative methyltransferase that is required for mitochondrial complex I function Journal of Cell Science 2010 123 10 1674 1683 10.1242/jcs.066076 20406883

[B32] UniProt CT Reorganizing the protein space at the Universal Protein Resource (UniProt) Nucleic Acids Res 2012 40 D71 D75 22102590 10.1093/nar/gkr981 PMC3245120

[B33] Logan DC Scott I Tobin AK The genetic control of plant mitochondrial morphology and dynamics The Plant Journal 2003 36 4 500 509 10.1046/j.1365-313X.2003.01894.x 14617080

[B34] Cox RT Spradling AC clueless, a conserved Drosophila gene required fro mitochondrial subcellular localization, interacts genetically with parkin Disease Models and Mechanisms 2009 2 9–10 490 499 19638420 10.1242/dmm.002378 PMC2737057

[B35] Motley AM Hettema EH Yeast peroxisomes multiply by growth and division The Journal of Cell Biology 2007 178 3 399 410 10.1083/jcb.200702167 17646399 PMC2064844

[B36] Dohm JA Lee SJ Hardwick JM Hill RB Gittis AG Cytosolic domain of the human mitochondrial fission protein fis1 adopts a TPR fold Proteins: Structure, Function, and Bioinformatics 2004 54 1 153 156 10.1002/prot.10524 PMC3047745 14705031

[B37] Motley AM Ward GP Hettema EH Dnm1p-dependent peroxisome fission requires Caf4p, Mdv1p and Fis1p Journal of Cell Science 2008 121 10 1633 1640 10.1242/jcs.026344 18445678 PMC2579327

